# Rectal laterally spreading tumors successfully treated in two steps by endoscopic submucosal dissection and endoscopic mucosal resection

**DOI:** 10.1186/1471-230X-10-135

**Published:** 2010-11-17

**Authors:** Italo Stroppa, Giovanni Milito, Raffaella Lionetti, Giovanni Palmieri, Federica Cadeddu, Francesco Pallone

**Affiliations:** 1 Gastrointestinal Unit, Department of Internal Medicine, Tor Vergata University, Rome, Italy; 2 Department of Surgery, Tor Vergata University, Rome, Italy; 3 Pathology Unit, Department of Biopathology, Tor Vergata University, Rome, Italy

## Abstract

**Background:**

Endoscopic submucosal dissection (ESD) is an advanced technique of therapeutic endoscopy alternative to endoscopic mucosal resection (EMR) for superficial gastrointestinal neoplasms >2 cm. ESD allows for the direct dissection of the submucosa and large lesions can be resected en bloc. ESD is not limited by resection size, increases histologically complete resection rates and may reduce the local recurrence.

Nevertheless, the technique is time-consuming, technically demanding and associated with a high complication rate. To reduce the risk of complications, different devices and technical advances have been proposed with conflicting results and, still, ESD en bloc resections of huge lesions are associated with increased complications.

**Case Presentation:**

We successfully used a combined ESD/EMR technique for huge rectal laterally spreading tumors (LSTs). ESD was used for circumferential resection of 2/3 of the lesion followed by piecemeal resection (2-3 pieces) of the central part of the tumour.

In all three patients we obtained the complete dissection of the polyp and the complete histological evaluation in absence of complications and recurrence at 6 months' follow up.

**Conclusions:**

In the treatment of rectal LSTs, the combined treatment - ESD/EMR resection may be considered a suitable therapeutic option, indicated in selected cases as an alternative to surgery, in which the two techniques are neither reliable nor safe separately. However, to confirm our results, larger trials with longer follow up are required together with improvement of the technique and of the technical devices.

## Background

Endoscopic mucosal resection (EMR) have become increasingly accepted and regularly used for the treatment of early gastric cancer and of superficial intestinal carcinomas with no risk of lymph node metastasis [1].

EMR can be performed using suction or lift technique such as EMRL/EMRC or EMR-strip biopsy [2-4]. Nevertheless, this method depends on the diameter of the lesions; if the neoplasm is larger than 2 cm, the method cannot always be feasible and is associated high recurrence rate, ranging between 2% and 35% [5].

Endoscopic submucosal dissection (ESD) is a new development in therapeutic endoscopy which allows for en bloc resection of sessile lesions regardless of the diameter, using special "devices" [6-8]. Briefly, the technique is performed as follows: 1) following 0,25% indigo carmine chromoendoscopy to evidence the margin of the tumour, the deeper submucosal tissue plane is opened using solutions such as saline, glycerol, sodium hyaluronate, or fructose; 2) the mucosa outside the markings is incised circumferentially using a flex-knife; 3) the connective tissue of the submucosal plane is dissected from the muscular layer [9].

However, this new technique, it is still associated with a higher incidence of complications than standard EMR procedure and requires a high level of endoscopic skill [10,11].

## Case Presentation

We herein report 3 cases of patients with rectal LSTs endoscopically treated at the Gastroenterology Unit of Tor Vergata University Hospital, Rome, between October 2006 and March 2007.

All patients presented with rectal LSTs larger than 3 cm (diameter was defined by positioning an open polypectomy snare close to the lesion); an en bloc resection was planned to ensure the accurate histopathologic assessment of the resected specimens and a sub-mucosal injection of hyaluronic acid was initially applied around the lesion. Nevertheless, in consideration of the impossibility to safety complete ESD, we decided to convert this technique into EMR- strip biopsy, performing a piecemeal resection of the centre of the polyps (two-three large pieces not less than 3 cm in diameter). Total procedure times were 45 minutes, 60 minutes, and 30 minutes respectively. All patients were hospitalized and discharged after 24-72 hours.

Only one intraoperative haemorrhage was observed. Given the ESD/EMR combined technique, patients were followed up at 3, 6 and 12 months for the first year then they were followed once a year.

### Patient 1

**Figure 1:**
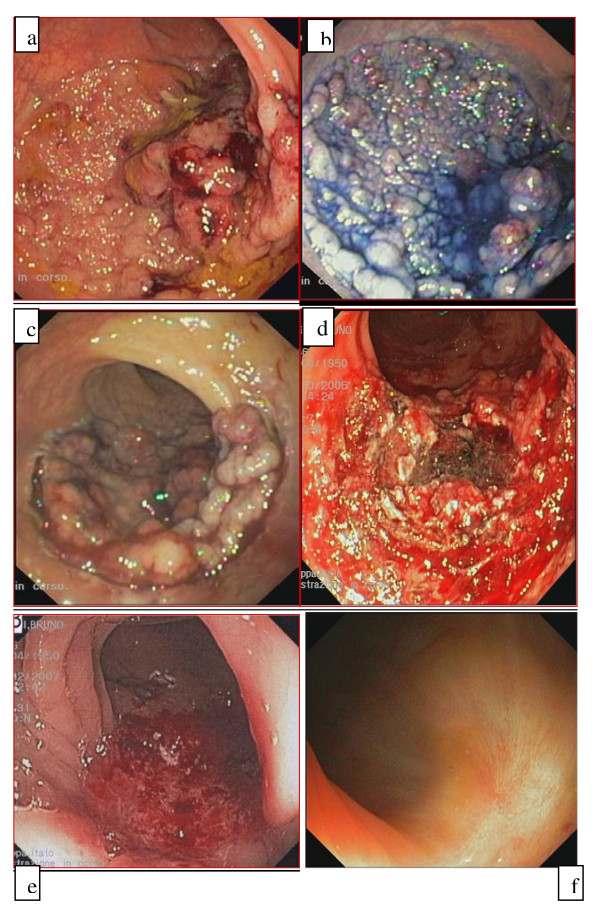
**Patient 1 ESD/EMR combined procedure**. **a **Detection of lesion. **b **Spraying with 1% indigo carmine. **c **Submucosal infiltration and lesion marking with Flex-Knife. **d-e **Removal of the lesion. **f **Endoscopic control at 3 months.

A 56-year-old male presented with a LST, 8-9 cm in diameter, located on the posterior wall of the rectum, 10 cm far from the anal verge and occupying 2/3 of the colonic lumen at colonoscopy (Figure 1a). The patient stated abdominal pain and lower gastrointestinal bleeding for 2 years.

Ecoendoscopy (Olympus GFUE160, Milan, Italy) showed a mucosal lesion with an area involving the superficial part of the submucosal layer (sm1), in absence of lymph node involvement.

The ESD/EMR combined procedure was carried out using a double-channel colonoscope (Olympus CF2T160I, Olympus, Milan, Italy) with the patient deeply sedated by propofol. Given the position and size of the lesion, a gastroscope (Olympus Gif Q 165, Olympus, Milan, Italy) retroflexed into the colon was used to resect the distal margin of the polyp.

The sub-mucosal injection of hyaluronic acid was performed around the lesion, previously enhanced with 1% indigo carmine (Figure 1b), followed by pre-cutting the surrounding mucosa of the tumor (Flex-knife Olympus, Tokio, Japan) (Figure 1c). The resection of the biggest part of the lesion was achieved using two knives (Hook-knife and insulation-tipped diathermic knife IT-knife; Olympus, Tokio, Japan). Nevertheless, even using a double-channel colonoscope (with the simultaneous use of forceps and knives), the excision of the central zone of the polyp was not completed. The remnant lesion was, therefore, removed by means of EMR-strip biopsy, with the "piecemeal" technique (Figure 1d). Post-operative course was uneventful. Histological examination revealed a tubulo-villous adenoma with high grade focal dysplasia. At 3 and 12 months' follow-up, no recurrence was detected in the excised area (Figure 1e, f).

### Patient 2

**Figure 2:**
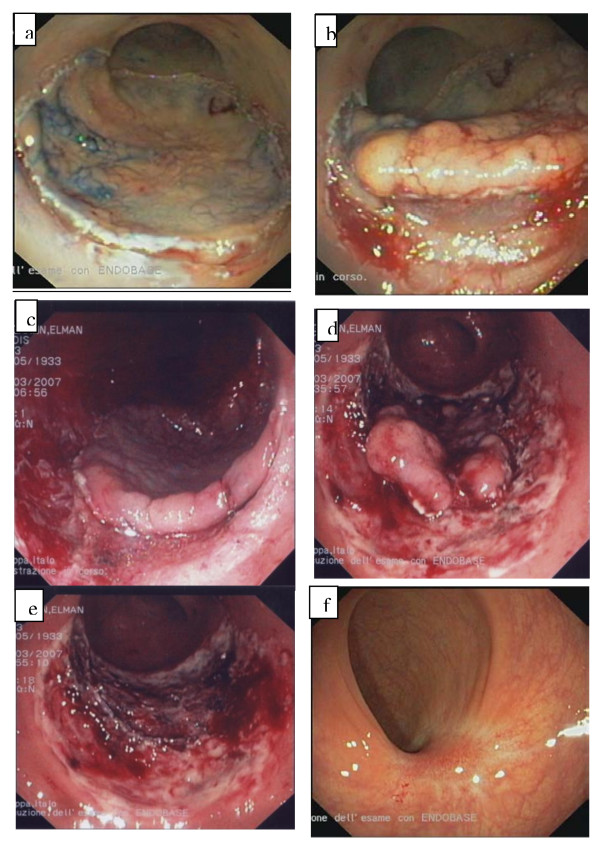
**Patient 2 ESD/EMR combined procedure**. **a **Spraying with 1% indigo carmine and lesion marking with Flex-Knife. **b **Infiltration of submucosa and removal of the anterior part of the lesion with Hook-Knife. **c **Anterior and lateral excision. **d **Removal of a large flap of lesion. **e **Complete polyp removal **f **Follow up at 6 weeks.

A 74-year-old male presented with a LST, 10 cm in diameter, located on the posterior wall of the rectum and 8 cm far from the anal verge at colonoscopy (Figure 2a). The patient stated haematochezia and mucorrea for 12 months. The eco-endoscopy excluded involvement of the submucosa,

ESD was planned; the polyp spray with 1% indigo carmine was carried out, followed by the injection of hyaluronic acid at the base of the lesion and by the pre-cutting of surrounding mucosa of the polyp with the Flex-Knife (Figure 2b).

The circumferentially excision was then performed, from the margin to the central area. Despite the use of dual channel colonoscopy, hook knife and repeated injections of hyaluronic acid, the complete en bloc resection was impossible due to the size of the lesion already excised (Figure 2c, d). The procedure was completed by EMR - strip biopsy- using the "piecemeal" technique (Figure 2e). Histological examination revealed tubulo-villous adenoma with low grade dysplasia. At 6 months' follow up endoscopy showed lack of recurrence (Figure 2f).

### Patient 3

A 59-year-old female presented with a LST approximately 8 cm in diameter, located at the recto-sigmoid junction at colonoscopy. The patient stated postdefecatory abdominal pain and rectal bleeding for few months. The ecoendoscopy, performed upon admission, excluded submucosa involvement.

**Figure 3:**
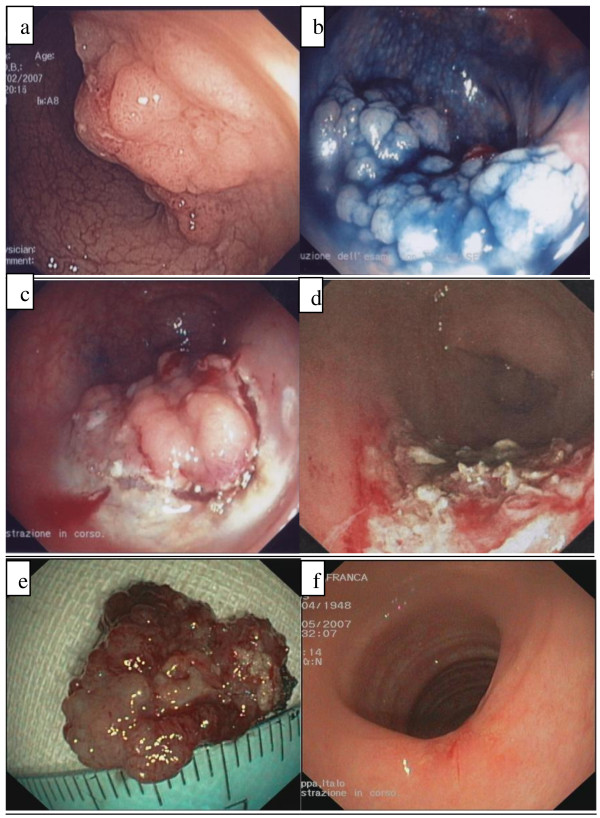
**Patient 3 ESD/EMR combined procedure**. **a **Endoscopic view of the polyp at the recto-sigmoid junction. **b **Spraying with 1% indigo carmine. **c **Infiltration of submucosa and anterior and lateral excision of lesion with Hook-Knife. **d **Removal of residual flap with complete removal of lesion **e **Fragment of excised lesion (2 cm in diameter) **f **Endoscopic control at 3 months.

ESD was planned (Figure 3a, b, c). ESD en bloc resection was not possible in consideration of the size and the site of the lesion. Thus, the resection was completed with EMR-strip biopsy, using piecemeal technique (Figure 3d), which allowed for complete removal (Figure 3e). During the procedure, a severe haemorrhage occurred at the base of the polyp and was successfully treated with the injection technique. At histology, a tubulo-villous adenoma with low grade dysplasia was detected. Three months' endoscopy showed intact mucosa in the procedure site (Figure 3f).

## Conclusions

EMR has been increasingly accepted for the treatment of gastrointestinal lesions at an early stage. Nonetheless, difficulty in correctly assessing the depth of tumour invasion and an increase in local recurrence have been reported for tumours larger than 2 cm, since such lesions are often resected piecemeal [12-14].

Furthermore, local recurrence after EMR is particularly difficult to treat, due to the submucosal fibrosis with an increased risk of complications [15-17].

ESD has been developed to remove the lesions in an en bloc fashion regardless of size, shape, coexisting ulcer, and location. ESD is, therefore, considered the treatment of choice to achieve en bloc resection of lesions > 2.0 cm and correct histological evaluation minimizing recurrence risk [18-20].

Although technically demanding, endoscopic submucosal dissection is quite effective and safe in experienced hands. Tanaka, Oka et al, in a study on 70 patients with LSTs successfully treated with ESD, reported 1.4% of cases of postoperative hemorrhage and 10.0% of cases of perforation. The rate of perforation markedly decreased with the practice of the technique. Moreover, the rate of perforation was high when an insulated-tip diathermic knife was used; practicing this technique was insufficient to reduce the rate of perforation. The average duration of follow-up was over 20 months, and no case of local persistence and recurrence or metastasis was observed [21].

The purpose of the present case series is to describe technical difficulties of ESD resection of rectal LST larger than 2 cm.

In all patients of our series ESD was planned, given the lesion size and depth. After the excision of the external part, we were unable to reach the centre of the LST removing the lesions en bloc [22]. This is due to the limited vision of the cutting area for huge lesions with difficulty in assessing the correct deeper submucosal tissue plane during the resection from the margins to the centre of the lesion.

Except for the excision of the distal part, achieved by a gastroscope retroflexed in the colon [23]. ESD treatment was performed with the dual channel colonoscope. The inconvenience of this device is the presence of both accessories emerging from the distal tract of the instrument, in a parallel vertical or transversal fashion, without the possibility of a co-ordinated action of the two equipments, effective both in lifting and cutting.

To solve this technical problem, in previous experiences we used other systems, most of which not successful, such as a 4.9 mm trans-nasal gastroscope and external grasping forceps parallel to the colonoscope [24], or clips to fix the excised part to the lesion still to be treated, or clips positioned below the excised flaps, acting as a "bridge" to reach the base of the lesion more easily. We also used the esophageal varices band ligator, without the straps, to make a dissection deeper than with the hood. An attempt was also made to position a polypectomy flap (25 mm wide and 50 mm long) on the residual base of the lesions, but the size of the previously excised flaps made correct placement of the snare for "en bloc" excision impossible.

**Figure 4:**
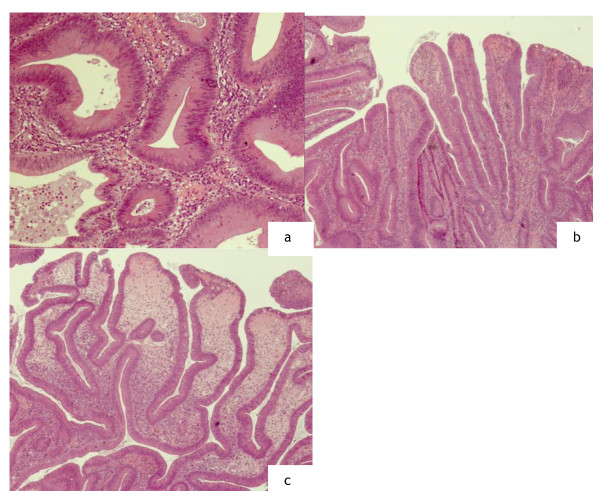
**Patient 4**. **a **Histological examination of the case 1 showing a tubulo-villous adenoma with high grade focal dysplasia. **b **microscopic view of the case 2 revealing tubulo-villous adenoma with low grade dysplasia. **c **histological examination of the patient **d **tubulo-villous adenoma with low grade dysplasia.

In our case series, ESD was planned and we subsequently decided to perform a combined ESD/EMR procedure considering both the polyp bulk and the limited number and the size of the fragments obtained from the "piecemeal" excision, suitable for correct histopathological evaluation (Figure 4a, b, c).

The accurate histopathologic assessment of the specimens is essential because the depth of the invasion and lymphovascular infiltration of the tumor is associated with considerable risk for lymph node metastasis. In our experience, no recurrence was detected during the follow up period.

Most possible node-negative epithelial neoplasms can be resected *en bloc *by ESD, when they are treated by very experienced hands. This does not mean that all endoscopic resection should be performed as ESD. Polypectomy or endoscopic mucosal resection (EMR) are beneficial for patients with pedunculated neoplasms, small neoplasms or large flat-elevated colorectal tumours because of the little invasiveness. If the lesions are apparently pre-malignant neoplasms, piecemeal resection by using EMR may be permissible with the best balance of risks and benefits [25].

Accordingly, Lishi and coworkers [26] removed 56 sessile colorectal polyps 2 cm or greater in diameter in 56 patients and reported a cure rate of 83% in patient submitted to piecemeal resection. They concluded that endoscopic piecemeal resection after submucosal saline injection with an intensive follow-up program is a safe and effective treatment for large, sessile colorectal polyps.

Therefore, although ESD en bloc resection, if possible, remains the treatment of choice [27,28] our experience shows the feasibility of this combined ESD/EMR treatment to overcome the difficulty in dissecting central area of massive lesions, also considering the absence of neoplastic recurrence at follow-up and the post-operative complications detected.

Accordingly Smith et al [29] stated the efficacy and safety of the combined ESD-EMR using the Olympus KD-630 L insulation-tipped knife even for the resection of an adenoma-like mass in chronic ulcerative colitis in 67 patients. The authors reported at a median of 18 months follow-up, an overall cure rates for the ESD-assisted EMR cohort of 98% Bleeding complications occurred in 7/67 (10%) of cases in absence of bowel perforations.

Besides this, a new double-channel endoscope ("R-scope") seems promising to solve this technical problem [30,31]; it allows for the vertical lifting of the mucosal layer with a forceps and the horizontal movement of a knife to cut the submucosal layer. Further studies are mandatory to confirm the validity of this new double-channel endoscope which would allow en bloc resection of complicated lesions on account not only of the large size, but also of the site, without ESD/EMR combined procedure.

In addition Uraoka and co-workers recently successfully treated 3 colorectal LSTs using the thin endoscope-assisted (TEA) ESD, a new traction system for improving submucosal cutting line visualization [32].

Hopefully, these devices will be forthcoming allowing to reach the central base of the lesions [31,33]. Meanwhile, this combined ESD/EMR treatment could be proposed for rectal giant polyps' resection, since it allows endoscopic removal of lesions reducing the risk of complications, particularly perforation and recurrence, considering the size and number of polyp fragments that allow for correct histopathological evaluation and low recurrence risk.

In conclusion, depending on the clinicopathological characteristics of the colorectal tumor, the colorectal ESD is indicated for lesions difficult to remove en bloc with a snare EMR [25]. In our experience, in the treatment of huge rectal polyps, shows that the combined treatment - ESD/EMR may be considered a suitable therapeutic option, indicated in selected cases as an alternative to surgery, in which the two techniques are neither reliable nor safe separately.

## Abbreviations

ESD: Endoscopic submucosal dissection; EMR: Endoscopic mucosal resection; LTS: laterally spreading tumors; TEA: thin endoscope-assisted.

## Competing interests

The authors declare that they have no competing interests.

## Authors' contributions

**IS**: manuscript preparation and critical review. **GM**: critical review. **RL**: data collection and manuscript preparation. **GP**: critical review. **FC**: literature review and manuscript preparation. **FP**: critical review. All authors read and approved the final manuscript.

## Pre-publication history

The pre-publication history for this paper can be accessed here:

http://www.biomedcentral.com/1471-230X/10/135/prepub

## References

[B1] Gotoda T Yamamoto H Soetikno RM Endoscopic submucosal dissection of early gastric cancer J Gastroenterol 2006 41 929 42 10.1007/s00535-006-1954-3 17096062

[B2] Karita M Tada M Okita K Kodama T Endoscopic therapy for early colon cancer: strip biopsy resection technique Gastrointest Endosc 1991 37 128 132 10.1016/S0016-5107(91)70669-X 2032596

[B3] Ponchon T Endoscopic mucosal resection J Clin Gastroenterol 2001 32 6 10 10.1097/00004836-200101000-00004 11154174

[B4] Higaki S Hashimoto S Harada K Nohara H Saito Y Gondo T Okita K Long-term follow up of large flat colorectal tumors resected endoscopically Endoscopy 2003 35 845 849 10.1055/s-2003-42622 14551863

[B5] Oka S Tanaka S Kaneko I Mouri R Hirata M Kanao H Kawamura T Yoshida S Yoshihara M Chayama K Endoscopic submucosal dissection for residual/local recurrence of early gastric cancer after endoscopic mucosal resection Endoscopy 2006 38 996 1000 10.1055/s-2006-944780 17058164

[B6] Watanabe K Ogata S Kawazoe S Watanabe K Koyama T Kajiwara T Shimoda Y Takase Y Irie K Mizuguchi M Tsunada S Iwakiri R Fujimoto K Clinical outcomes of EMR for gastric tumors: historical pilot evaluation between endoscopic submucosal dissection and conventional mucosal resection Gastrointest Endosc 2006 63 776 782 10.1016/j.gie.2005.08.049 16650537

[B7] Onozato Y Ishihara H Iizuka H Sohara N Kakizaki S Okamura S Mori M Endoscopic submucosal dissection for early gastric cancers and large flat adenomas Endoscopy 2006 38 980 986 10.1055/s-2006-944809 17058161

[B8] Imagawa A Okada H Kawahara Y Takenaka R Kato J Kawamoto H Fujiki S Takata R Yoshino T Shiratori Y Endoscopic submucosal dissection for early gastric cancer: results and degrees of technical difficulty as well as success Endoscopy 2006 38 987 990 10.1055/s-2006-944716 17058162

[B9] Lee I-L Lin PY Tung S-Y Shen CH Wei KL Wu CS Endoscopic submucosal dissection for the treatment of intraluminal gastric subepithelial tumors originating from the muscularis propria layer Endoscopy 2006 38 1024 1028 10.1055/s-2006-944814 17058168

[B10] Kakushima N Fujishiro M Kodashima S Muraki Y Tateishi A Omata M A learning curve for endoscopic submucosal dissection of gastric epithelial neoplasms Endoscopy 2006 38 991 995 10.1055/s-2006-944808 17058163

[B11] Fujishiro M Yahagi N Kakushima N Kodashima S Muraki Y Ono S Kobayashi K Hashimoto T Yamamichi N Tateishi A Shimizu Y Oka M Ogura K Kawabe T Ichinose M Omata M Successful nonsurgical management of perforation complicating endoscopic submucosal dissection of gastrointestinal epithelial neoplasms Endoscopy 2006 38 1001 1006 10.1055/s-2006-944775 17058165

[B12] Hurlstone DP Sanders DS Cross SS Adam I Shorthouse AJ Brown S Drew K Lobo AJ Colonoscopic resection of lateral spreading tumors: a prospective analysis of endoscopic mucosal resection Gut 2004 53 1334 1339 10.1136/gut.2003.036913 15306595 PMC1774165

[B13] Saito Y Fujii T Kondo H Mukai H Yokota T Kozu T Saito D Endoscopic treatment for laterally spreading tumors in the colon Endoscopy 2001 33 682 86 10.1055/s-2001-16213 11490384

[B14] Tanaka S Haruma K Oka S Takahashi R Kunihiro M Kitadai Y Yoshihara M Shimamoto F Chayama K Clinicopathological features and endoscopic treatment of superficial spreading colorectal neoplasms larger than 20 mm Gastrointest Endosc 2001 54 62 66 10.1067/mge.2001.115729 11427843

[B15] Hurlstone DP Sanders DS Cross SS George R Shorthouse AJ Brown S A prospective analysis of extended endoscopic mucosal resection for large rectal villous adenomas: an alternative technique to transnasal endoscopic microsurgery Colorectal Dis 2005 7 339 44 10.1111/j.1463-1318.2005.00813.x 15932555

[B16] Walsh RM Ackroyd FW Shellito PC Endoscopic resection of large sessile colorectal polyps: technical implications and results over eight years Dis Colon Rectum 1986 29 831 35 10.1007/BF02555357 3792164

[B17] Seewald S Ang TL Omar S Groth S Dy F Zhong Y Seitz U Thonke F Yekebas E Izbicki J Soehendra N Endoscopic mucosal resection of early esophageal squamous cell cancer using the Duette mucosectomy kit Endoscopy 2006 38 1029 1031 10.1055/s-2006-944527 17058169

[B18] Fujishiro M Yahagi N Omata M Successful outcomes of a novel endoscopic treatment for GI tumor: endoscopic submucosal dissection with a mixture of high-molecular-weight hyaluronic acid, glycerin, and sugar Gastrointest Endosc 2006 63 243 249 10.1016/j.gie.2005.08.002 16427929

[B19] Yokoi C Gotoda T Hamanaka H Oda I Endoscopic submucosal dissection allows curative resection of locally recurrent early gastric cancer after prior endoscopic mucosal resection Gastrointest Endosc 2006 64 212 218 10.1016/j.gie.2005.10.038 16860071

[B20] Fujishiro M Endoscopic submucosal dissection for stomach neoplasm World J Gastroenterol 2006 12 5108 5112 16937520 10.3748/wjg.v12.i32.5108 PMC4088006

[B21] Tanaka S Oka S Kaneko I Endoscopic submucosal dissection for colorectal neoplasia: possibility of standardization Gastrointestinal Endoscopy 2007 66 100 107 10.1016/j.gie.2007.02.032 17591481

[B22] Repici A Conio M De Angelis Sapino A Malesci A Arezzo A Hervoso C Pellicano R Comunale S Rizzetto M Insulated-Tip Knife endoscopic mucosal resection of large colorectal polyps unsuitable for standard polypectomy Am J Gastroenterol 2007 102 1 7 10.1111/j.1572-0241.2007.01198.x 17403075

[B23] Hurlstone DP Sanders DS Thomson M Cross SS "Salvage" endoscopic mucosal resection in the colon using a retroflexion gastroscope dissection technique: a prospective analysis Endoscopy 2006 38 902 906 10.1055/s-2006-944733 16981107

[B24] Imaeda H Iwao Y Ogata H Ichikawa H Mori M Hosoe N Masaoka T Nakashita M Suzuki H Inoue N Aiura K Nagata H Kumai K Hibi T A new technique for endoscopic submucosal dissection for early gastric cancer using an external grasping forceps Endoscopy 2006 38 1007 1010 10.1055/s-2006-925264 16673308

[B25] Fujishiro M Perspective on the practical indications of endoscopic submucosal dissection of gastrointestinal neoplasms World J Gastroenterol 2008 14 4289 4295 10.3748/wjg.14.4289 18666315 PMC2731178

[B26] Iishi H Tatsuta M Iseki K Narahara H Uedo N Sakai N Ishikawa H Otani T Ishiguro S Endoscopic piecemeal resection with submucosal saline injection of large sessile colorectal polyps Gastrointest Endosc 2000 51 697 700 10.1067/mge.2000.104652 10840302

[B27] Reavis KM Melvin WS Advanced endoscopic technologies Surgical Endoscopy 2008 22 1533 46 10.1007/s00464-008-9831-1 18401657

[B28] Kakushima N Fujishiro M Endoscopic submucosal dissection for gastrointestinal neoplasms World J Gastroenterol 2008 14 2962 7 10.3748/wjg.14.2962 18494043 PMC2712159

[B29] Smith LA Baraza W Tiffin N Cross SS Hurlstone DP Endoscopic resection of adenoma-like mass in chronic ulcerative colitis using a combined endoscopic mucosal resection and cap assisted submucosal dissection technique Inflamm Bowel Dis 2008 14 1380 6 10.1002/ibd.20497 18465807

[B30] Yonezawa J Kaise M Sumiyama K Goda K Arakawa H Tajiri H A novel double-channel therapeutic endoscope ("R-scope") facilitates endoscopic submucosal dissection of superficial gastric neoplasms Endoscopy 2006 38 1011 1015 10.1055/s-2006-944779 17058166

[B31] Neuhaus H Costamagna G Devière J Fockens P Ponchon T Rösch T (ARCADE Group) Endoscopic submucosal dissection (ESD) of early neoplastic gastric lesions using a new double-channel endoscope (the "R-scope") Endoscopy 2006 38 1016 1023 10.1055/s-2006-944830 17058167

[B32] Uraoka T Kato J Ishikawa S Harada K Kuriyama M Takemoto K Kawahara Y Saito Y Okada H Thin endoscope-assisted endoscopic submucosal dissection for large colorectal tumors Gastrointestinal Endosc 2007 66 836 9 10.1016/j.gie.2007.04.028 17905031

[B33] Akahoshi K Honda K Akahane H Akiba H Matsui N Motomura Y Kubokawa M Endo S Higuchi N Oya M Endoscopic submucosal dissection by using a grasping-type scissors forceps: a preliminary clinical study Gastrontest Endosc 2008 67 1128 1133 10.1016/j.gie.2007.12.007 18355820

